# Investigating an e-cigarette brand’s use of music festivals for social media campaigns and experiential marketing

**DOI:** 10.18332/tpc/199607

**Published:** 2025-02-03

**Authors:** George D. H. Pearson, Diana L. Davidson, Barbara A. Schillo, Jennifer M. Kreslake

**Affiliations:** 1 Schroeder Institute, Truth Initiative, Washington, United States

**Keywords:** e-cigarettes, advertising, experiential marketing, social media, festivals, Geek Bar

## Abstract

Geek Bar is an e-cigarette brand that has seen a substantial rise in sales in the past year. During this time, the brand has utilized an extensive experiential marketing campaign targeting music and arts festivals, especially electronic dance music (EDM) events. This campaign utilizes both festival attendance and social media to create associations between the brand and EDM culture. To analyze this campaign, we downloaded and coded 18 months of Instagram posts by Geek Bar for the festivals mentioned and relevant themes. During the last six months of data collection, 39% of all Instagram posts by Geek Bar were about festivals. The account regularly drew connections between the brand and EDM culture, showing artists/DJs performing alongside young, attractive e-cigarette users. Such attempts to create ties between the brand and EDM culture are concerning given the younger age of EDM fans. These techniques will likely continue due to the lack of regulations and enforcement on e-cigarettes and experiential marketing.

## INTRODUCTION

Geek Bar is an emerging e-cigarette brand, having risen from less than 2% of the US market share at the start of 2024 to 15.6% by September 2024^1^. This made it the third top selling e-cigarette brand^2^, and it was predicted to overtake JUUL in sales by the end of 2024^1^. Of youth e-cigarette users, 5.8% reported using Geek Bar in 2024^3^. The brand’s rapid sales increase coincides with a social media campaign and experiential marketing focusing on connecting Geek Bar to music and arts festivals, especially electronic dance music (EDM) events.

Tobacco companies have a long history of co-opting cultural events via sponsorships^4-6^. These tactics make consumers associate products with fun and excitement ‘and as a means of expressing rebellion and independence’^4^. Attending EDM festivals specifically is likely to be of concern as EDM fans generally tend to be younger in age^7-9^.

E-cigarette brands are not prohibited from attending or sponsoring events such as festivals, and they have leveraged experiential advertising tactics in the past^5^. Notably, concerts were identified as a key part of JUUL’s youth targeting^10^. However, in 2024, 86% of e-cigarette sales in the US were for illegal products^11^. This includes Geek Bar, which does not have the required marketing authorization from the FDA and thus cannot be legally marketed in the U.S.

Using Python scripts, automated browsers using the Selenium and Playwright packages scraped links and then downloaded content from all posts on the @geekbarvape Instagram account over an 18-month period (June 2023 to November 2024). Posts not viewable when logged out of Instagram (n=2) were not included, leaving n=558 posts. Downloaded posts were hand-coded by two authors for relevancy to festivals. Festival relevant posts were then coded by two authors for: the specific festival mentioned; showing use of the product; showing music performers; attempts to tie Geek Bar to the festival experience (e.g. telling followers to ‘get ready to elevate their festival experience with Geek Bar’ or describing Geek Bar as ‘the ultimate music festival sidekick’); and solicitation of engagement (e.g. posts about contests or asking questions such as ‘Which flavor was your festival go-to? Drop it in the comments’).

## COMMENTARY

**Figure 1:**
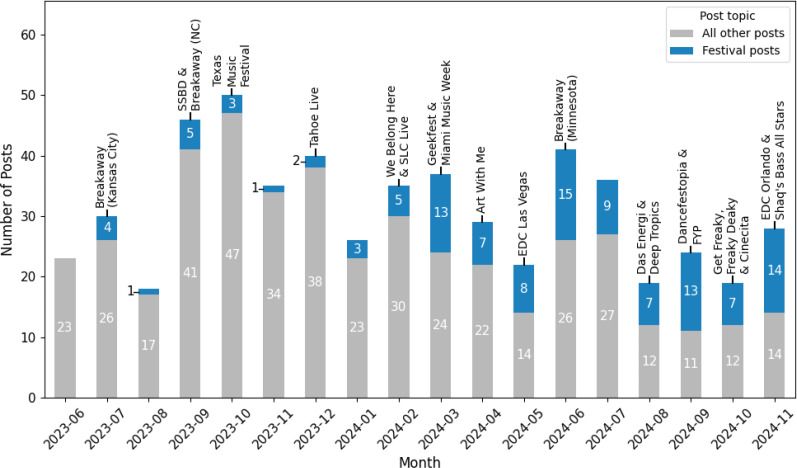
Bar graph showing the total number, and number of festival-related, posts per month with dates of festivals mentioned

Figure 1 shows the number of posts made each month and the number about festivals. Of 558 posts, 117 (21%) featured festivals. However, the frequency of festival-related posts has grown. We compared three 6-month periods (June to November 2023, December 2023 to May 2024, June to November 2024). The first six months saw 14 of 202 posts about festivals (6.9%), the second 38 of 189 (20.1%), and the third 65 of 167 (38.9%). Proportions z-tests showed the second period had a greater proportion of festival posts than the first (z=3.83, p<0.001); and the third period had a greater proportion than the first (z=7.46, p<0.001) and second periods (z=3.907, p<0.001).

Eighteen different festivals were referenced. These include GeekFest, an EDM event in Dallas created and hosted by Geek Bar, described as ‘a symphony of Vape and Vibes’. Electronic Daisy Carnival (EDC) Las Vegas was the most mentioned festival (n=25), followed by GeekFest (n=14), and the Breakaway series of festivals (n=10). The majority were EDM festivals and all featured EDM acts. However, some had additional focusses, for instance, Geek Bar attended Deep Tropics, which describes itself as a place ‘where music, art, style, and sustainability converge’^12^.

Many posts connected use of Geek Bar with the festival experience (n=40), for instance stating ‘Geekbar Meloso is here to turn up the vibes and make your night unforgettable’, or telling users to ‘get ready to vape to the rhythm’. While many posts showed festival attendees using the e-cigarettes (n=35), many focused on the festival, showcasing DJs and artists performing (n=34).

Solicitation of engagement was also used (n=27). For instance, one post told users that they could win tickets to EDC Las Vegas by tagging a friend in the comments and sharing the post with a specified hashtag. Another told users to share their ‘most epic 360 selfie’ taken at Geek Bar’s festival booth.

The account also highlighted festival booth attractions, showing pictures of people posing with e-cigarettes, Geek Bar plush pillows, or using a rotating selfie photo booth. Example posts can be seen in Supplementary file Figure 1. Pictures often focused on festival attendees, with numerous photos featuring attractive young people posing with e-cigarettes. One video also advertised 1000 people had sampled Geek Bar e-cigarettes at one festival, likely contravening US federal law^13^. Other posts had minimal connection to e-cigarettes directly. For instance, the account posted daily countdowns to festivals Geek Bar would be attending.

Experiential marketing has become a major part of Geek Bar’s content on Instagram. Over the course of six months 39% of posts by @geekbarvape were about festival appearances, making it the dominant theme. These posts routinely drew connections between EDM culture and Geek Bar, either via text descriptions or images, and videos of artists and DJs performing alongside young, attractive e-cigarette users.

Given EDM music generally attracts a younger audience, often in their early twenties^7-9^, campaigns targeting festival attendees are more likely to attract new nicotine users rather than those switching from combustible tobacco. Furthermore, the social media campaign that focuses on the exciting imagery and music of the festivals alongside young, attractive attendees is especially likely to be appealing to youth^14-16^.

### Limitations

This study is only observational. We make no causal claims between Geek Bar’s marketing and sales, and claims about marketing strategies are only inferred from social media content. Additionally, we analyzed one social media account. Different strategies may be used on other platforms or media.

## CONCLUSION

Experiential marketing has been an effective technique for the tobacco industry^4,5^. While cigarette and smokeless tobacco brands have some restrictions on utilizing experiential marketing, e-cigarette brands do not face the same restrictions and have used them to attract young users in the past^10^. Geek Bar, however, is not authorized for marketing or sale in the United States^3^, meaning attendance and marketing at US festivals may be prohibited. Still, they have continued to leverage these tactics.

## Supplementary Material



## Data Availability

The data supporting this research are available from the authors on reasonable request.
