# Supplementary material for: Investigating an e-cigarette brand’s use of music festivals for social media campaigns and experiential marketing
Source: Tob Prev Cessat. 2025 Feb 3;11:10.18332/tpc/199607. doi: 10.18332/tpc/199607 (PMC11788851; doi:10.18332/tpc/199607)
Supplement: Supplementary file 1 [file TPC-11-10-s1.pdf]

Figure 1.

Sample posts by @geekbarvape Instagram account.

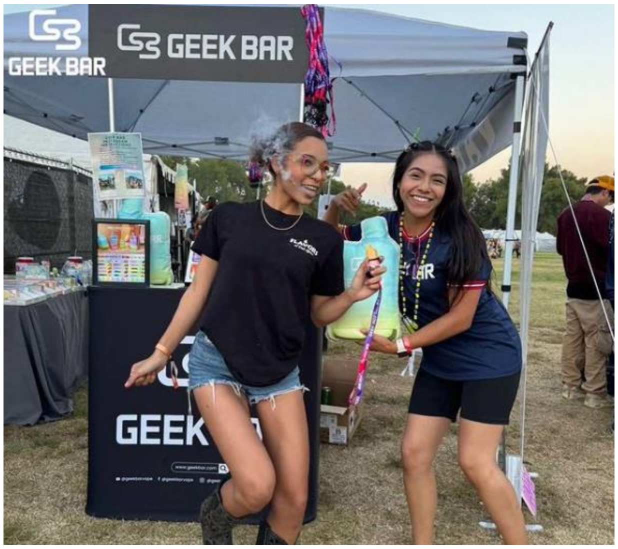

Posted September 30th, 2023: “The magic of music meets the innovation of Geekbar Meloso - this same same but different music festival @ssbdfest is bound to be unforgettable!”

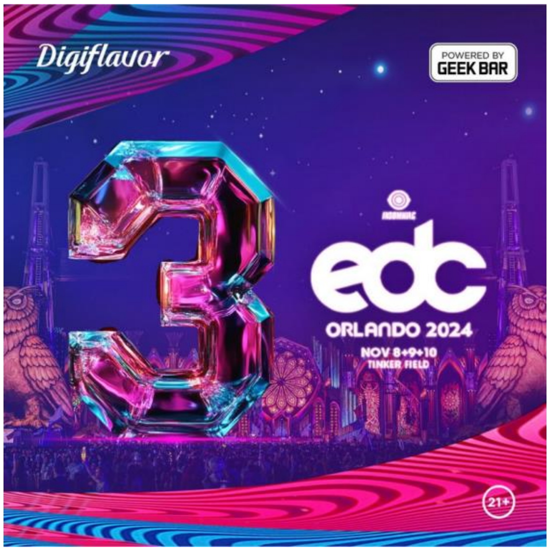

Posted November 6th, 2024: “Meet Digiflavor & GEEKBAR at EDC Orlando #EDCLV #geekbar #musicfestival #EDM #edcorlando”

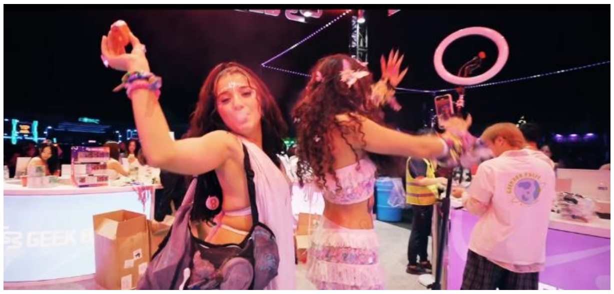

Screenshot of video posted June 12th, 2024: “Feel the excitement! The GeekBar & EDC Las Vegas aftermovie is here. Share your favorite scene with us!”

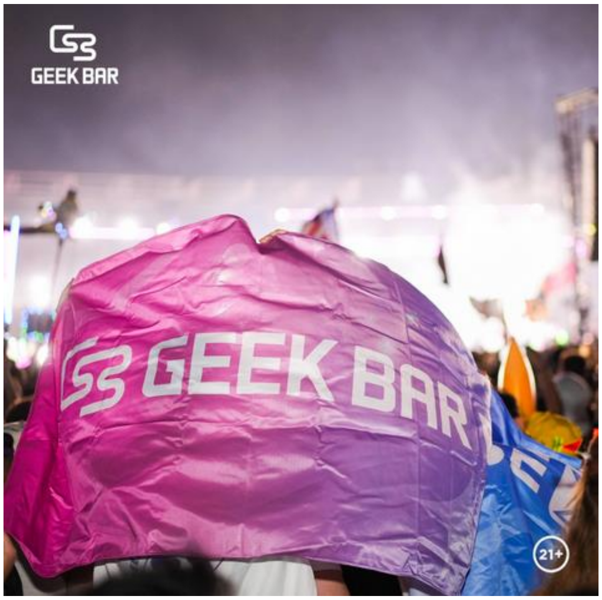

Posted 6th July 2024: “Share your best GeekBar moment at EDC Las Vegas!”

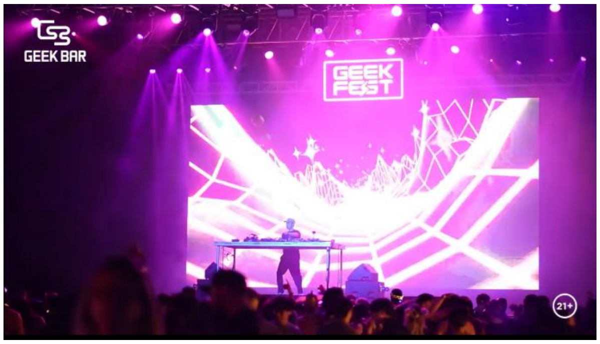

Screenshot of video posted March 27, 2024: “Immerse yourself in the dynamic vibes of Geekfest! Our event video transports you to the heart of the action, showcasing the high-energy atmosphere and captivating performances that lit up the night at the music festival in America!”

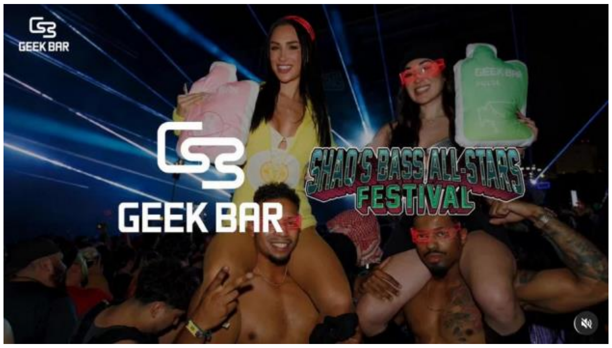

Screenshot of video posted 29th November 2024: “Geekbar video from Shaq's Bass All-Stars Festival ✨ 🎵🎶 big bass 🤯 loads of high energy dancing fun!! #GEEKBAR #geekbar #Geekbar #GeekBar #vapecommunity #ShaqsBassAllStars #FestivalVibes #musicfestival”

© 2025 Pearson G.D.H. et al.
